# Pupils’ Acceptance and Plate Waste of Sorghum-Based Breakfasts in South African School Feeding Programmes: A Mixed-Methods Study Across Five Provinces

**DOI:** 10.3390/ijerph23020192

**Published:** 2026-01-31

**Authors:** Hema Kesa, Eridiong Onyenweaku, Alex Dimitri Tchuenchieu Kamgain

**Affiliations:** 1 Food Evolution Research Laboratory, School of Tourism and Hospitality, University of Johannesburg, Johannesburg 2092, South Africa; eridiongo@uj.ac.za; 2 Centre for Food, Food Security and Nutrition Research, Institute of Medical Research and Medicinal Plants Studies, Yaoundé 13033, Cameroon

**Keywords:** sorghum, plate waste, school feeding, breakfast, South Africa

## Abstract

**Highlights:**

**Public health relevance—How does this work relate to a public health issue?**

- This study targets South Africa’s National School Nutrition Programme (NSNP), which is a key public health intervention reaching vulnerable school-aged children.
- Focusing on learner acceptance and plate waste, the study links meal consumption behaviours to the effectiveness of the school feeding programme in delivering intended nutritional and educational benefits.

**Public health significance—Why is this work of significance to public health?**

- The findings highlight a global acceptance of sorghum-based foods, which are of high interest for the NSNP.

**Public health implications—What are the key implications or messages for practitioners, policy makers, and/or researchers in public health?**

- Improving sensory appeal and serving conditions may significantly reduce plate waste and enhance learner intake.

**Abstract:**

Sorghum-based porridges are a key component of breakfast meals in South African school feeding programmes. While these meals support learner nutrition and educational outcomes, their effectiveness depends on learner acceptance and the extent of plate waste. This study assessed acceptance and plate waste of two sorghum-based porridges—Mabele (100% sorghum) and Morvite (pre-cooked sorghum, 75–100% depending on flavour, with possible inclusion of soya, cow’s milk, and wheat/gluten)—compared with instant maize meal, Jungle Oats (100% wholegrain oats), within the Tiger Brands Foundation breakfast programme. Patterns of waste and underlying reasons were examined across five provinces. A mixed-methods approach was used in 25 primary schools across Gauteng, KwaZulu-Natal, Limpopo, North West, and Northern Cape. Quantitative data were collected through 10-day food waste diaries completed by Volunteer Food Handlers and analysed using descriptive statistics, ANOVA, and regression models. Qualitative data were obtained from 75 semi-structured staff interviews and 25 learner focus groups, analysed thematically using ATLAS.ti version 22. Overall, food waste was low, with “no food waste” reported in over half of the observations. Acceptance of sorghum-based products varied. Morvite was generally well accepted, whereas Mabele was frequently disliked in some provinces. Key drivers of waste included food dislike, poor preparation, bland flavour, and learner absenteeism, with serving conditions and a lack of utensils as secondary factors. Although waste was modest, variability in acceptance of sorghum-based porridges suggests the need to improve preparation quality, flavour, and serving conditions to enhance programme effectiveness.

## 1. Introduction

By definition, school feeding programmes (SFPs) are designed in many countries to provide children with at least one nutritious meal per day (breakfast, lunch, or both) while they are in attendance [1]. Those programmes are commonly implemented to address child poverty, food insecurity, and malnutrition [2]. It was estimated in 2022 that SFPs were helpful to approximately 418 million children worldwide who received free or subsidised meals [3]. This is of high importance considering that according to global burden of disease estimates, hundreds of millions of children continue to experience various forms of malnutrition, highlighting persistent high levels of nutritional risk especially in low- and middle-income regions [4].

In South Africa, a governmental SFP known as the National School Nutrition Programme (NSNP) is implemented. It was formerly called the Primary School Nutrition Programme (PSNP), which was initiated in 1994 to improve developmental outcomes among disadvantaged children [5]. Reported benefits include improved concentration, school attendance, and overall well-being of pupils. Breakfast provision has been linked to better cognitive performance, memory, and classroom participation [6,7]. A recent report indicated the country as one of the 20 nations responsible for 65% of all children experiencing severe food poverty worldwide. In South Africa alone, 23% of children are still vulnerable to malnutrition and associated health risks [8]. This matter of poverty and hunger among children is therefore still a challenge.

The Tiger Brands Foundation (TBF) has supported the NSNP by providing in-school breakfasts since 2011. As part of this partnership, the TBF supplies four breakfast porridges to selected schools, among which are sorghum-based products of different flavours and other products like Ace Instant (maize-made) and Jungle Oats. However, the effectiveness of breakfast programmes relies greatly on pupils’ willingness to eat the meals provided and on how much of the food is actually consumed rather than discarded. Indeed, sorghum is certainly recognised as an indigenous South African food, but a recent study among South African adults have shown very low knowledge and consumption [9,10]. This staple grain is yet well known for its high nutritional value (including its fiber content, micronutrient profile, and potential for sustained energy release) as well as its health benefits [11,12], making sorghum-based porridges a valuable option for school meal programmes but which might be rejected by pupils.

Plate waste represents an indicator of the effectiveness of school feeding programmes [13]. A high waste also reduces the nutritional benefit intended for pupils and results in financial losses and environmental burdens. Previous research in South Africa has examined nutritional quality and operational performance within the NSNP [5,14,15], providing valuable insights into the programme management. However, limited evidence exists on learner acceptance and plate waste specifically related to sorghum-based breakfast porridges. This study addresses this gap by examining pupils’ acceptance and plate waste of sorghum-based breakfasts within the Tiger Brands Foundation programme across five provinces.

## 2. Materials and Methods

### 2.1. Study Design

A mixed qualitative and quantitative research approach was used to assess pupils’ acceptance of four porridge products provided by the Tiger Brands Foundation to support the South African National School Feeding Programme. The products included Ace Instant Porridge (instant maize meal), Jungle Oats (100% wholegrain oats), Mabele (100% sorghum), and Morvite (pre-cooked sorghum, 75–100% depending on the flavour, which may contain soya, cow’s milk, and wheat/gluten). More detailed product descriptions are available at https://www.tigerbrandsfoodservicesolutions.com (accessed on 3 December 2025).

The qualitative approach was used to fully understand the feelings and opinions of participants through interviews and focus group discussions. The quantitative aspect employed food waste diaries to assess the preference of pupils in the schools. This mixed-method approach was chosen to triangulate acceptance and waste patterns and therefore bring out reliable conclusions.

### 2.2. Location of the Study and Target Population

The research was conducted across five provinces in specific geographical regions: Gauteng (urban), KwaZulu-Natal (rural), North West (peri-urban), Limpopo (peri-urban), and Northern Cape (peri-urban and rural areas). A total of 5 primary schools were selected in each province, resulting in 25 schools overall. The schools were chosen from a pre-existing database provided by Tiger Brands Foods, which included only primary schools participating in the breakfast programme.

The target population consisted of educators and staff, NSNP coordinators/teaching assistants, School-based Volunteer Food Handlers (VFH), and volunteer pupils from Grades 5 to 7.

### 2.3. Sampling Procedure and Data Collection

#### 2.3.1. Qualitative Data

At each school, individual interviews were conducted with an educator/NSNP coordinator, the Principal/Head of Department were interviewed, along with a VFH responsible for meal preparation and service. This yielded a total of 75 interviews across the 25 schools.

Focus group discussions (FGDs) were conducted with pupils from Grades 5, 6, and 7. A total of 4 pupils per grade participated, resulting in 12 pupils per school and 25 FGDs overall. The FGDs explored pupils’ understanding of food waste and porridge preferences.

Data were collected using a self-developed interview questionnaire. The guide included open-ended questions exploring the frequency of food waste and the reasons for food waste in the school feeding context. The semi-structured format ensured consistency across schools while allowing participants to elaborate on their experiences. Interviews and FGDs were audio-recorded with consent and transcribed verbatim for analysis. The interviewers were trained before going in the field for data collection.

#### 2.3.2. Quantitative Data

Food waste diaries were completed by VFHs or NSNP coordinators in each school for 10 consecutive school days (2 weeks). The diaries captured daily observations of leftover porridge quantities for each food item. This was helpful to estimate the percentage of schools reporting food waste for each of the 04 breakfast meals compared.

Food waste was not captured at the individual pupil level but at the school meal level, based on food remaining after service and collected waste from pupils’ plates. Food waste was recorded separately for each breakfast product (Ace Instant, Jungle Oats, Mabele, and Morvite), using distinct containers or waste bags. Only one breakfast product was served per day, following the NSNP weekly rotation, allowing for waste to be attributed to a specific product. Each of the 04 breakfast products was served at least 02 times per week in each of the schools. Daily records were aggregated per school over the 10-day period and then summed at the provincial level to generate the percentage of reported waste cases. No individual-level weighting was applied.

During the FGDs conducted in each school, the 12 pupils were invited to raise their hands if they disliked each of the four breakfast products cited consecutively. Additional explanations were provided when necessary to ensure that all participants clearly understood the products being discussed. This approach made it possible to identify unpreferred breakfast items at the provincial level (60 pupils: 12 pupils × 5 schools) and subsequently later in the 05 targeted South African provinces.

### 2.4. Data Analysis

Qualitative data from interviews and FGDs were transcribed verbatim and analysed on ATLAS.ti^®^ version 22 using an inductive thematic analysis approach. Themes emerged directly from participants’ narratives without imposing predefined categories, ensuring that pupils’ and staff perspectives were accurately represented.

Quantitative data from food waste diaries were analysed using descriptive and inferential statistics. ANOVA was used to compare differences in food waste across the four porridge items, while multinomial logistic regression was performed to examine variation in waste across provinces “no food waste” as the reference category.

### 2.5. Ethics

Ethical approval for the study was obtained from the Research Ethics Committee of the College of Business and Economics at the University of Johannesburg, with the ethics clearance code 24STH12. Permission to access the schools and conduct the study was obtained from the Provincial Department of Education. Caregivers, children, food handlers, school nutrition coordinators, and principals were fully informed of the study’s purpose and procedures and were assured of their right to withdraw at any time. Their consents were obtained before they could take part to the study. No personal identifying information was collected, and confidentiality was maintained throughout.

## 3. Results

### 3.1. Food Waste

**Figure 1:**
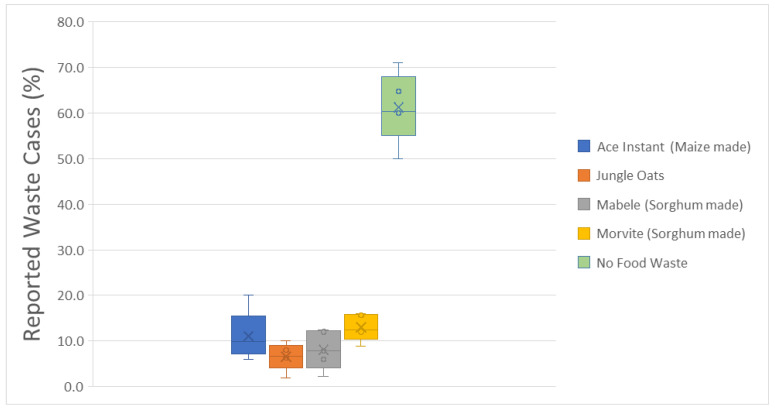
Overall food wastage among the studied schools over the observation period.

The overall food wastage among the studied schools is illustrated by Figure 1. Most of the schools (61.2 ± 7.7%) reported no food waste over the 10-day observation period. Nevertheless, non-negligible levels of food item waste were also recorded. Morvite appeared as the most reported wasted food item (13.0 ± 2.9%), followed by Ace Instant (11.0 ± 5.4%) and Mabele (8.1 ± 4.3%). Jungle Oats was the least cited (6.6 ± 3.0%). However, the observed differences within the food item waste cases were not statistically significant.

**Figure 2:**
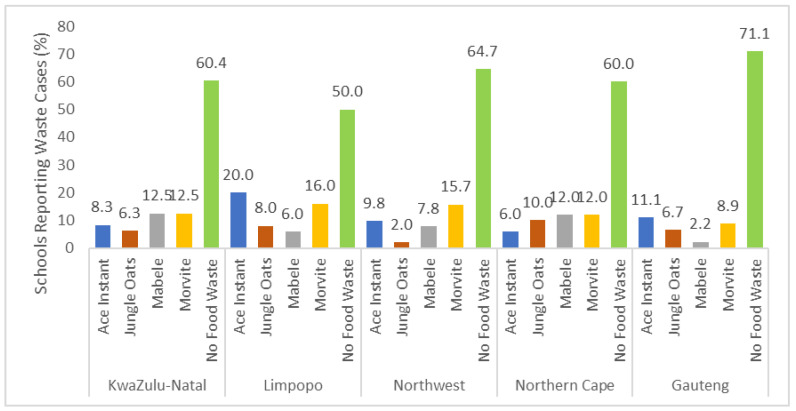
Regional distribution of schools reporting waste cases during the observation period.

Figure 2 provides the percentage of food wastage cases across each of the five provinces, as well as the common food items that are wasted. Gauteng province had the lowest food wastage, with 71.1% of respondents reporting no food wastage. In contrast, Limpopo province was the province with the highest wastage (50%). At first glance, the distribution of waste cases across food items varied with the province. Ace Instant (maize-made) showed considerable variation, with the highest percentage in Limpopo (20.0%) and lower values in provinces such as the Nortwhern Cape (6.0%). Jungle Oats remained relatively low across provinces, reaching its highest value in the Northern Cape (10.0%) but dropping to 2.0% in the North West. For Mabele (sorghum-made), the percentages were moderately distributed, with the KwaZulu-Natal and Northern Cape both reaching 12.5% and 12.0%, whereas Gauteng showed the lowest proportion (2.2%). Morvite (sorghum-made) showed moderate levels of waste cases, ranging from 8.9% in Gauteng to 16.0% in Limpopo. The statistical analysis of this data (Table 1) also revealed no significant difference in the waste cases associated to the food items in the different provinces.

Table 2 presents breakfast meals that pupils in the five provinces (North West to Limpopo) find unpreferred. Each row represents a type of breakfast meal, such as Jungle Oats, Instant Porridge, and others, while the columns show the number of pupils expressing dislike out of the total of 60 pupils who participated in the FGDs in each province. Ace Instant Porridge stands out as the most unpreferred meal overall, with 56 indicating dislike out of the total of 300 pupils across the five provinces, particularly in Gauteng (21) and Limpopo (22). Mabele was the second most unpreferred, with 47 out of the total 300 participating pupils, and it is especially unpopular in Northern Cape, where 28 reported disliking it. Morvite, which like Mabele is made from sorghum, was disliked by only 8 pupils out of the total 300, a number close to the only 4 pupils who reported disliking Jungle Oats (for which pupils had a clear preference across the provinces).

### 3.2. Frequency of Food Waste and Reasons

**Figure 3:**
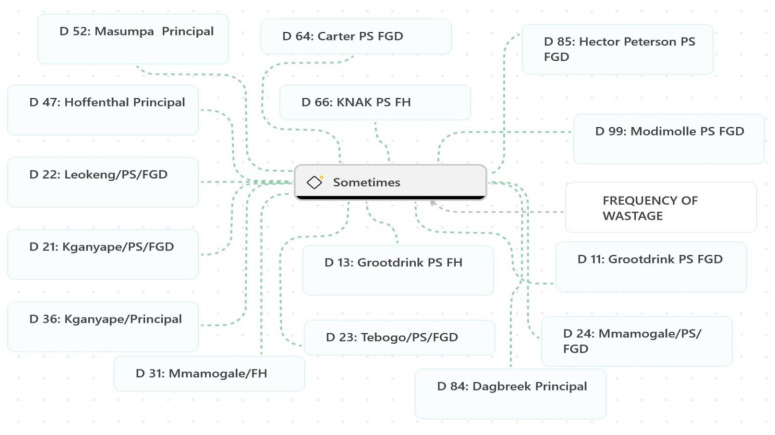
Key word characterising frequency of food waste.

The frequency of food wastage was quite often described by participants as occurring “sometimes,” (Figure 3) suggesting an irregular but present issue across various contexts and provinces. For instance, a learner from a primary school in Northern Cape noted, “We just eat a bit and sometimes when we don’t finish it”, highlighting partial consumption as a recurring cause of wastage. Similarly, a learner from the same school responded to the question “Do we waste food?” with “Sometimes”. Similar responses were reported in primary schools in the North West province. When asked whether food was thrown away, a learner admitted, “Ah, sometimes,” while participants from another school in the same province used comparable expressions, including “Sometimes.” A consistent perspective was also shared in a different primary school in the North West, where a participant again stated, “Sometimes.” Principals across different schools echoed these observations. For example, a Principal in KZN stated, “Ooh, sometimes they leave the food”. Similarly, another Principal in KZN acknowledged, “Yah, that is why sometimes we do have like uh little bit of waste”. While the irregularity of wastage was emphasised by some, such as in a primary school in Gauteng where it was observed, “It’s just sometimes but not every day”, this intermittent occurrence was evident across most schools and provinces. Participants from a primary school in Limpopo admitted to occasional wastage, with a learner responding “Yes, sometimes” when asked about food waste. Similarly, in another school in Limpopo, a learner remarked, “Sometimes we throw it away”. These responses as shown in Figure 3, collectively illustrate that while food waste may not be a constant issue, it persists sporadically across schools in various provinces, and this causes some level of concern.

**Figure 4:**
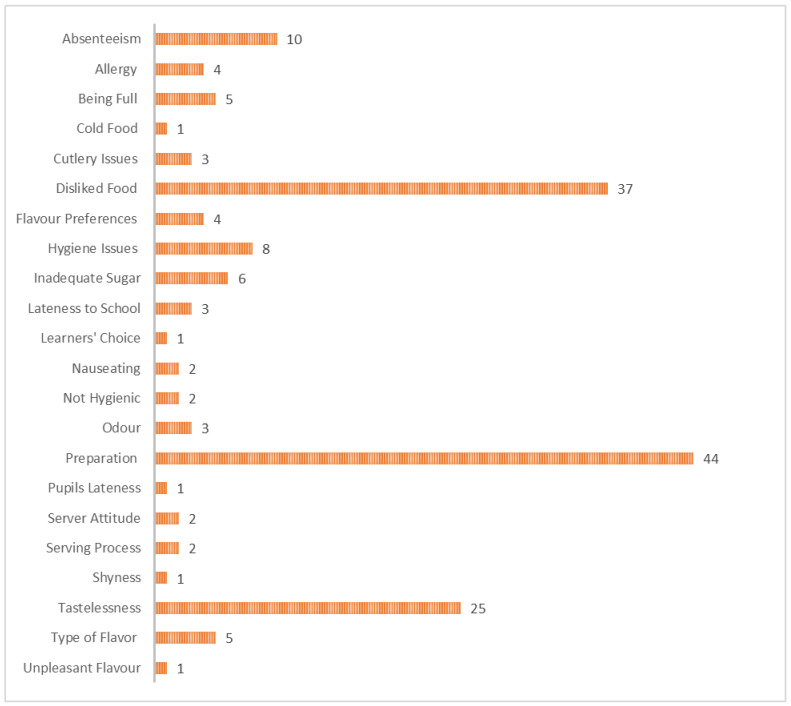
Reported reasons for food waste and the number of times each was cited.

Figure 4 presents the most common causes of food waste during breakfast that were reported during the interviews. The main reasons were poor preparation, pupils’ dislike of the food, perceptions of tastelessness, and student absenteeism.

- Preparation of Food—Participants highlighted concerns about how food was prepared, noting issues with texture, consistency, and cooking methods. In Northern Cape, some felt the food lacked care, with one coordinator stating, “*Sometimes it’s not prepared nicely*”. Similarly, in North West, participants also explained that “*Sometimes it’s burnt*,” while others added, “*It’s like they are forced to cook, they just don’t want to*,” and emphasised the frustration of finding “*raw sorghum, you can even feel it*.” In a school in Limpopo, pupils complained that “*They pour a lot of water in the instant porridge*,” while others said, “*Sometimes it’s dry like braai pap*,” reflecting inconsistencies in food preparation.
- Disliked Food—Dislike of certain foods is one of the most frequently cited reasons for food waste. A coordinator explained, “*Sometimes they don’t like certain porridges… so that day they always waste it*” (NC). A Principal noted, “*Most of the kids don’t like Ace instant*” (LP). These examples illustrate how pupils’ preferences can result in certain foods being discarded.
- Tastelessness—The perceived blandness of the food was a recurring issue, often linked to a lack of sugar. In Northern Cape, pupils from a school repeatedly emphasised, “*It’s sour. It’s sour*,” while the Principal from a different school explained, “*They are used to sugar at home, so they tend to shy away when there’s no sugar*.” Similar statements were expressed in KZN, where some pupils complained, “*It has very little sugar*,” and a student stated, “*My least favourite food is sorghum because it doesn’t taste good*.”
- Absenteeism—Absenteeism is a significant contributor to food waste in schools. When pupils are absent, the prepared food often goes uneaten. For example, a coordinator noted, “*When children… are absent… there will be waste*” (NC). Similarly, another Principal explained, “*Most learners are absent on that day per class, then that’s where the food is wasted*” (LP). A Principal also shared, “*Let’s say for example, learners are forty but you find that only thirty-five came to school. Yah, so you see some food will be wasted*” (GP). These remarks underscore how absenteeism directly leads to surplus food and waste.

A few other secondary reasons were also reported as it can be seen in that Figure 3. They include the following:

- Food Allergy—Food allergies are another cause of food waste, as some children cannot consume certain meals. A participant remarked, “*Fish, the problem with fish is some children are allergic*” (LP). Additionally, pupils in a school expressed discomfort: “*It gives me stomach pains*” (KZN) and “*It gives me a runny tummy*” (KZN). These allergies often result in uneaten meals, contributing to food waste.
- Being Full—Some children arrive at school already full, having eaten at home, which leads to food waste. A coordinator observed, “Some kids come full to school because they ate at home” (NC). It was also noted in a distinct school, “*It happens sometimes that the children eat breakfast at home, and when they get here, they are full*” (KZN). These instances show how food prepared at school goes uneaten when pupils have already eaten at home.
- Cold Food—The texture and temperature of food play a role in waste. A participant commented, “*Why not mabele guys? Sometimes when it gets cold, it wobbles*” (LP). This shows that cold or unappealing textures deter pupils from eating, contributing to food wastage.
- Cutlery Issues—The lack of proper cutlery or clean utensils also leads to food waste. A learner in a school shared, “*They give us porridge with plates that are wet*” (GP), while another noted, “*Because they don’t give me a spoon*” (GP). These practical challenges prevent pupils from eating their meals, resulting in wasted food.
- Flavour Preferences—Pupils’ preferences for specific flavours contribute to food waste. A Principal remarked, “*It’s just a matter of comparing the flavors*” (FS). Similarly, at another primary school, it was noted, “*Learners have favourite meals… Like on Monday, it’s Instant, and most of them don’t eat Instant*” (LP). This selective eating leads to waste when meals are not to pupils’ liking.
- Hygiene Issues—Concerns about hygiene are another factor contributing to food waste. A learner mentioned, “*The people that pour the porridge do not wash their hands*” (GP). In a different school, pupils reported seeing “*cockroaches*” and even a “*worm*” in the food (GP). These hygiene concerns discourage pupils from eating, leading to significant waste.
- Pupils’ Lateness—Late arrivals were also cited as a reason some children missed meals, with one participant in the North West explaining, “*Sometimes it is because they are late.*” This problem compounded the frustrations around accessing properly cooked food.
- Servers’ Attitude—The attitude of servers significantly influenced pupils’ experiences. In the North West, pupils from a school shared that “*They dish up for us in anger*,” and added, “*When someone does not serve us food from a good heart, we get worried*.” These feelings impacted how children perceived the meals and reflected an emotional barrier to enjoying the food provided.
- The Serving Process—Long queues and rushed service were frequent complaints, especially in the North West, where pupils of a school noted, “*Sometimes the line is very, very, very long.*” A participant from another school highlighted issues with the serving process, saying, “*When they dish up mabele during break time, they don’t remove the hard top layer*.” These experiences contributed to dissatisfaction with the overall meal experience.
- Shyness among Pupils—In Gauteng, a Principal observed that “*Some learners are afraid of eating in front of their friends, especially in grade seven*,” illustrating the social pressures that sometimes discourage older pupils from participating in school meals.
- Type of Flavour—Participants expressed preferences for certain flavours while disliking others. In the North West, a learner simply stated, “*Strawberry*,” as a preferred choice, while a learner from another school said, “*I just don’t like the banana flavor because I grew up eating plain white porridge*.” Not all flavours were welcomed, particularly the strawberry flavour, which some found off-putting. In Gauteng, a Deputy Principal explained, “Sometimes they don’t prefer the strawberry flavor because it’s red-ish in colour.” This demonstrates how visual and sensory cues influenced meal acceptance.

**Figure 5:**
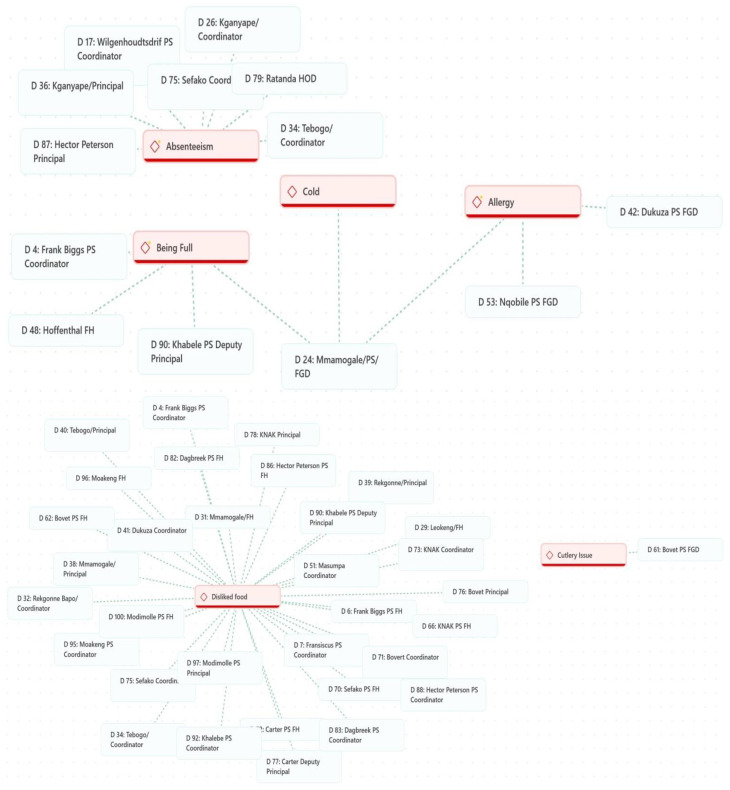
Reasons for food waste at the schools according to schools’ teams.

Globally, school teams interviewed particularly pointed out food dislike and pupils’ absenteeism as the main reasons of the food wastes as illustrated in Figure 5.

## 4. Discussion

This research study examined pupils’ acceptance of four porridge products provided by the TBF and the extent and determinants of plate waste within South African school breakfast programmes across five provinces. Two of the products were sorghum-based (Mabele and Morvite), one was maize-based (Ace Instant), and the last was made from oats (Jungle Oats).

### 4.1. Acceptance of Sorghum-Based Breakfast Meals

Sorghum is a nutrient-dense cereal, providing 7–12% protein and a rich source of dietary fiber and essential minerals like zinc, iron, and calcium [11,12,16]. This makes it adequate for school meals, also considering its affordability and cultural relevance in parts of South Africa. The study results revealed that pupils’ acceptance of sorghum-based porridges varied considerably. Mabele was widely unpreferred, particularly in the Northern Cape, whereas Morvite was comparatively well accepted and showed low reported waste. These contrasting outcomes underscore that sorghum acceptance is not uniform and might depend on several factors, such as flavour, texture, added ingredients, and preparation consistency. Indeed, according to the product descriptions provided online by the TBF, Mabele consists solely of unmilled or finely milled sorghum grains, yielding a robust texture and earthy flavour suited for traditional porridges. On the other hand, Morvite is produced from pre-cooked sorghum (75–100%) and may also contain soya, cow’s milk, and wheat/gluten depending on the flavour. This difference in formulation could have contributed to the observed variation in the preferences. This aligns with previous studies indicating that acceptance of indigenous grains among children is often conditioned by sensory attributes and habitual exposure rather than the grain itself. Studying the important sensory attributes affecting consumer acceptance of sorghum porridge in West Africa, Aboubacar et al. [17] noticed that the textural characteristics of stickiness in the mouth and cohesiveness were the most important sensory attributes, followed by the taste and aroma of the product.

### 4.2. Food Waste Patterns and Drivers

Most pupils described food waste as occurring “sometimes,” reflecting that waste is not persistent but rather context-dependent. Quantitative findings showed no significant differences in waste across the four products and across provinces. Qualitative findings offer deeper insight into the drivers of waste. Across schools, the most frequently cited reasons for waste included food dislike, poor preparation, the lack of flavour, and absenteeism. Meal acceptance is strongly influenced by the sensory properties of food and the consistency of preparation [18,19]. Issues such as burnt porridge, insufficient cooking, incorrect dilution, and a lack of sugar directly shaped perceived palatability. For pupils accustomed to sweetened or richly textured home-prepared porridges, the school versions might have often been considered bland, contributing to waste. The level of food waste is influenced, among other factors, by students’ perceptions and acceptance of school meals, as well as their eating habits at home [20]. Mumba & Kesa [21] had already identified portion size and poor delivery services as causes of food waste in the NSNP. Absenteeism also emerged as an important structural factor. Where pupils were absent on particular days (due to school schedules, transport challenges, or personal reasons), prepared food remained uneaten, inflating overall waste levels. This suggests that real-time adjustments could help reduce discarded portions.

### 4.3. Contextual and Environmental Contributors

Several secondary reasons for waste such as a lack of clean cutlery, cold meals, insect contamination, server attitudes, or long queues highlight the importance of the school food environment in shaping consumption behaviours. Mafugu [22] had already pointed out poor kitchen facilities as one of the South African School Nutrition Programme constraints. Even when pupils enjoy a particular food item, negative experiences related to serving conditions may discourage consumption. These findings reinforce the need to consider the entire service chain (from product delivery to preparation, plating, and hygiene) when designing strategies to reduce waste. Physical and social factors also played a role. Some pupils, particularly in senior primary grades, reported shyness about eating in front of peers, which may reflect informal social norms around school meals. Similarly, pupils who arrived at school already full or who had eaten at home tended to waste more food. This tendency had already been reported by Blondin et al. [23] when studying food waste in a universal free school breakfast program. These behavioural patterns echo broader research demonstrating that school feeding programmes succeed best when integrated into household food routines and when school-level social dynamics are considered [24].

### 4.4. Limitations of the Study

This study relies on self-reported waste diaries, which may be influenced by reporting errors or variations in how VFHs interpret “waste”. Nonetheless, the triangulation of data sources strengthens confidence in the overall patterns observed. Socio-demographic characteristics of participants (e.g., age, gender, and household income) were not collected, although these factors may influence meal acceptance and food wastage. This was due to time and logistical constraints within the school setting. Future studies should incorporate these variables to better account for potential confounding effects.

## 5. Conclusions

While food waste in the school breakfast programme of South Africa is generally modest, key challenges remain, particularly related to the acceptability of certain porridge types and the quality of preparation. Sorghum-based products hold significant nutritional interest, but improvements in formulation, flavour, preparation, and serving conditions are essential for enhancing learner acceptance. Addressing both preference-related and structural contributors to waste can improve the effectiveness of school nutrition programmes, reduce resource loss, and support better learner nutrition outcomes.

## Figures and Tables

**Table 1:** Likelihood of food item waste across provinces.

Variable		Odd Ratio (OR)	Confidence Interval (95%CI)		*p*-Value
			Min	Max	
Ace Instant	Gauteng	1.03	0.27	3.91	0.96
	KwaZulu-Natal	0.91	0.22	3.71	0.90
	Limpopo	2.64	0.80	8.70	0.11
	Northern Cape	0.66	0.15	3.00	0.59
	North West	1.00			
Jungle Oats	Gauteng	3.09	0.31	31.32	0.34
	KwaZulu-Natal	3.41	0.34	34.65	0.30
	Limpopo	5.28	0.56	50.20	0.15
	Northern Cape	5.50	0.61	49.80	0.13
	North West	1.00			
Mabele	Gauteng	0.26	0.03	2.43	0.24
	KwaZulu-Natal	1.71	0.44	6.65	0.44
	Limpopo	0.99	0.20	4.83	0.99
	Northern Cape	1.65	0.42	6.42	0.47
	North West	1.00			
Morvite	Gauteng	0.52	0.14	1.88	0.32
	KwaZulu-Natal	0.85	0.26	2.75	0.79
	Limpopo	1.32	0.44	4.00	0.62
	Northern Cape	0.82	0.26	2.65	0.75
	North West	1.00			

**Table 2:** Student-reported unpreferred meal per province.

Meal	Province					Total (/300)
	Gauteng (/60)	KwaZulu-Natal (/60)	Limpopo (/60)	North West (/60)	Northern Cape (/60)	
Ace Instant	21	5	22	7	1	56
Jungle Oats	0	0	4	0	0	4
Mabele	2	5	7	5	28	47
Morvite	2	0	1	5	0	8

## Data Availability

All relevant data are within the paper. However, if additional information is required, it will be provided upon request from the corresponding author.
